# Challenges and opportunities in generating and utilizing environmental data for sustainable food systems

**DOI:** 10.1002/jeq2.70269

**Published:** 2026-09-27

**Authors:** J. L. Taylor, J. Tzilivakis

**Affiliations:** ^1^ Agriculture and Environment Research Unit (AERU), School of Health, Medicine and Life Sciences University of Hertfordshire Hatfield UK

## Abstract

Modern agricultural production has detrimental effects on the environment including contributing toward current global climate and biodiversity crises. It is therefore necessary that our agroecosystems, and wider food systems, become sustainable, so that the growing population can be fed now and in the future. Agroecosystems are complex with multiple inputs, outputs and interactions, with consequent environmental effects and impacts. This complexity is mirrored in an equally complex “data ecosystem” that is utilized by different stakeholders to make decisions. New understanding and technologies are driving a data revolution. Data on the environmental impacts and performance of food production systems have undoubtedly grown in recent years, and we have a better understanding and more data on causal activities, environmental effects, and outcomes. However, we still often lack what is needed to provide a reliable and holistic picture of the environmental impacts of food production (covering all impacts on the atmosphere, biosphere, hydrosphere, and lithosphere), and a common picture of our food system to ensure that different stakeholders and their actions are pulling in the same direction. This is a complex topic with many scientific, technological, commercial, and social challenges. However, these also present opportunities to advance our understanding of environmental systems and identify the real changes needed to address sustainability issues. This paper explores these challenges and opportunities and their potential to facilitate a transition to sustainable food systems. It highlights that although many challenges remain, there are opportunities for real change to address food security and sustainability issues.

## Core Ideas

- Food systems need to become sustainable to address current environmental crises.
- Agroecosystems are complex, and this complexity is mirrored in an equally complex data ecosystem that is utilized by different stakeholders to make decisions.
- Data to provide a reliable and holistic picture of the environmental impacts of food production are often lacking.
- This is a complex topic with many scientific, technological, commercial, and social challenges.
- These are opportunities to advance our understanding of environmental systems and identify the real changes needed to address food security and sustainability issues.

## Plain Language Summary

Farming and environmental systems are complex, with multiple inputs, outputs, and interactions, with consequent environmental effects and impacts including climate change and damage to wildlife populations. This complexity is mirrored in an equally complex data ecosystem that is utilized by different people to make decisions. New understanding and technologies are driving a data revolution but, despite this growth in data, we still often lack what is needed to provide a reliable and full picture of the environmental impacts of food production. This is a complex topic with many scientific, technological, commercial and social challenges. This paper provides a broad overview of current challenges and opportunities in the production of environmental data, how it is handled, and its utilization to enable a transition to sustainable food systems. It highlights that although many challenges remain, there are opportunities for real change to address food security and sustainability issues.

**Abbreviations**
AI: artificial intelligence
CAP: Common Agricultural Policy
CO_2_e: carbon dioxide equivalent
eDNA: environmental DNA
GHG: greenhouse gas
LCA: Life Cycle Assessment
PEF: Product Environmental Footprint

## 1 INTRODUCTION

Modern agriculture has brought about huge change to how we farm, what we farm and where we farm. From the early work on breeding and genetics by Gregor Mendel and others, to Norman Borlaug and the green revolution in the 1960s, our ability to grow better crops and animals, and in much larger quantities, has increased. This has kept the global population fed but now needs to increase further with estimates of the global population reaching ∼10 billion by 2050. However, many of the advances made in agriculture, such as artificial fertilizer inputs, use of pesticides, as well as antibiotics use in animals, are having a detrimental effect on the environment both directly and indirectly, including contributing toward current global climate (IPCC, 2023; Lynch et al., 2021; Zurek et al., 2022) and biodiversity crises (Dasgupta, 2021; IPBES, 2019). It is therefore necessary that our agroecosystems, and wider food systems, become sustainable, so that the growing population can be fed now and in the future.

Agroecosystems are complex and dynamic with flows of materials and energy, chemical, biological, and social interactions, and different farming systems and practices that have variable effects and impacts in different locations. The complexity of agroecosystems is mirrored in an equally complex “data ecosystem” that attempts to provide a picture to different end users. These data ecosystem often forms the interface between socio‐ecological stakeholders operating in the same space. It encompasses ecological, sociocultural, technological, economic, and political dimensions of food systems from production to consumption. To understand which practices are having negative and positive impacts on the environment, and the significance of these with respect to sustainability, environmental data need to be collected for a wide range of metrics encompassing activities, effects, and outcomes. These data can then be utilized along the supply chain for different end purposes. A fundamental question is whether the environmental data ecosystem is fit for purpose to aid the development of sustainable food systems. The challenge is to present a reliable and consistent picture to all stakeholders (e.g., data generators, processors, consumers, and many others) that tells the same story across the food system so that common sustainability goals can be achieved. The transition of agroecosystems needs to be coupled with an evolution in our data ecosystems to facilitate the development of sustainable food systems, with data‐based solutions being recognized as key assets to boost the resiliency and sustainability of food systems (HECPAD, 2023; Šipka & Stagianni, 2024).

The demand for environmental data in the food system is growing (Figure 1). In the past, the demand for environmental data was largely confined to government departments, agencies, and regulators, to gain a picture of the pressures, state and responses to environmental issues to help steer government policies and initiatives. However, the number and diversity of end users demanding environmental data has grown in recent years, and this is only going to increase in the future (e.g., to meet Scope 3 greenhouse gas [GHG] emissions reporting). Figure 1 shows the diversity of purposes and end users of environmental data. Some of these will be single‐issue focused (e.g., climate change/GHG emissions, or biodiversity), while others aim to cover a range of environmental issues. This issue alone creates scope for externalization of impacts. However, it is further compounded by using different data, metrics, calculations, and interpretations of the same issues. For example, in Figure 1, farmers may collect data on activities (e.g., livestock numbers, practices, and methods of production) to calculate GHG emissions using a carbon calculator to help identify hotspots for mitigation. Food manufacturers may also calculate GHG emissions for their products, but based on secondary data that may not account for method of production, thus can be very different to those at the farm level. Carbon emissions is one of the more established issues with respect to data and metrics available. Data associated with other issues, such as biosphere‐related impacts (e.g., measures of biological diversity, species populations, habitat suitability, etc.), are more variable and/or missing. This all creates further scope for differing and contradictory pictures resulting in barriers to consensus and, in a worst‐case scenario, making the wrong decisions.

**FIGURE 1:**
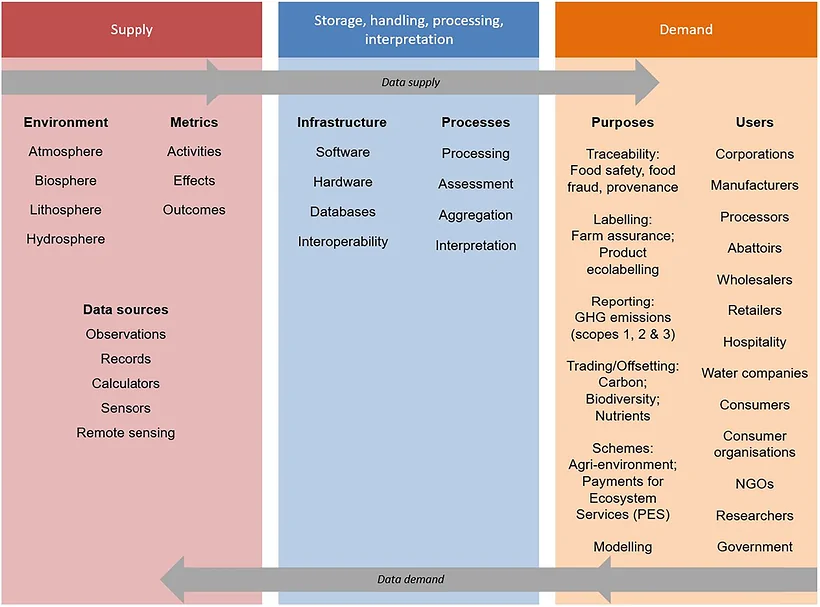
The data ecosystem: Supply and demand of environmental data.

With respect to the transition to sustainable food systems, we need to know when the adoption of practices that improve environmental performance deliver the outcomes society desires. This demands a reliable picture of the environmental impacts of food production, and the data ecosystem needs to reflect that reliable picture: data and metrics used to build or represent that picture must reflect changes at the farm level. The danger is that if different methods and/or metrics are used for different schemes and/or different purposes, we can end up with what Tzilivakis et al. (2024) have termed “perspective disparity,” that is, we have different pictures of the same system. Additionally, when taking the more holistic perspective needed to facilitate the evolution of sustainable food systems, in some instances, parts of the picture may be unclear or completely missing. Thus, there is a fundamental risk of having a wrong or incomplete picture. For example, Antonelli et al. (2024) highlight how some carbon trading schemes focus on maximizing removal of CO_2_ from the atmosphere, with options such as the planting of eucalyptus trees in African savannahs, which can result in harming biodiversity. These issues potentially create conflicting drivers in the system that are not conducive with achieving progress toward sustainability goals and potentially drive the system in the wrong direction (Béné et al., 2019; Brouwer et al., 2020; Bustamante et al., 2024).

We need harmonization across the data ecosystem to avoid conflicting pictures and ensure all parts are pulling the same direction (with respect to achieving sustainability), and we need a more complete (holistic) view to ensure that all elements needed for sustainability are included and not externalized from decision‐making processes. This sounds fine in theory, but in practice, it is complex with significant challenges to overcome. Data that are fit for purpose for one use‐case may not be for another, but it is still essential to avoid contradictory or conflicting pictures. For example, there should be a common environmental picture for a farm participating in a government agri‐environment scheme (Defra, 2024a), an offsetting scheme for biodiversity (Defra, 2013; Millon et al., 2021), and an ecolabel for its products (FE, 2024); that is, they all synergistically reinforce each other with respect to environmental goals. However, an alternative scenario is a farm in an agri‐environment scheme that does not qualify for the biodiversity offsetting scheme, as the metrics are incompatible with offsetting schemes (Defra, 2024b), and the biodiversity benefits achieved from the agri‐environment scheme are not represented within the ecolabel (Tzilivakis et al., 2024).

**Core Ideas**

- Food systems need to become sustainable to address current environmental crises.
- Agroecosystems are complex, and this complexity is mirrored in an equally complex data ecosystem that is utilized by different stakeholders to make decisions.
- Data to provide a reliable and holistic picture of the environmental impacts of food production are often lacking.
- This is a complex topic with many scientific, technological, commercial, and social challenges.
- These are opportunities to advance our understanding of environmental systems and identify the real changes needed to address food security and sustainability issues.

This is a complex topic, and although there have been many advances in recent years, many challenges remain including scientific, technological, commercial, and social challenges. This paper aims to provide an overview of the challenges and opportunities that arise in the production of environmental data, handling the data produced, and its utilization within the context of enabling a transition to sustainable food systems.

## 2 CHALLENGES

Challenges fall into two main areas. First, our scientific understanding in terms of what we can measure and what methods are available to reliably determine the health of the environment and the sustainability of our food systems. Second, what can be realistically undertaken with respect to environmental monitoring and data collection, accounting for technological, commercial (practicality and cost), and social constraints.

### 2.1 Scientific, measurement, and interpretation challenges

#### 2.1.1 Scientific understanding

A fundamental challenge is ensuring we have sufficient scientific understanding and data on environmental issues, not just in terms of the state of the environment and the damage being done, but also the causal activities, processes, and mechanisms, using appropriate research tools and methods to provide accurate information. Without this, making decisions that facilitate an evolution toward sustainable food systems is difficult. In parallel to this, we also need robust and reliable metrics that will provide the data to quantify and qualify the picture. We cannot measure everything everywhere all the time, so we are often reliant on indicators and models, which will vary with respect to their reliability. Different approaches can help our overall understanding of some issues, but there is scope for different metrics to present different pictures of the same thing. Additionally, how data and metrics are interpreted within the context of their use is also critical not only with respect to understanding environmental impacts and outcomes but also communicating these to relevant stakeholders and decision‐makers.

Our scientific understanding of the environmental impacts of food production has grown substantially in recent decades. This includes the sources and conditions under which pollution occurs, the environmental damage caused by agricultural activities, and the state of the environment. However, there are still many aspects that are not fully understood both in terms of the extent of environmental damage and/or the causes of that damage. For example, declines in biodiversity and wildlife species populations are often attributed to “intensive agriculture” (Chiffoleau et al., 2024; Raven & Wagner, 2021), but the specific causes are often unclear, are highly complex, involving multiple causal factors, or can be bespoke to individual species. For example, some species require specific habitats to be present within a given range to complete their lifecycle; if one or more are missing, then the landscape will not be suitable for that species. Habitat connectivity and habitat complementarity issues such as this can be crucial factors for some species but are highly complex and in many instances not fully understood. Such gaps in knowledge and understanding present a key challenge for the transition to sustainable food systems where both future research and practical applications are needed.

#### 2.1.2 Metrics and perspective breadth

Metrics can be broadly categorized into those which are a measure of environmental outcomes (endpoints), those which cover environmental effects (midpoints), and those which cover causal activities and processes. This echoes the pressure‐state‐response framework (and variations thereof) presented by the OECD in the 1990's (OECD, 1993). Measures of activities and practices tend to be the easiest to measure, followed by midpoint effects and impacts, with measures of environmental outcomes (endpoints) being the most difficult or costly to measure. In other instances, we have robust measures of the state of the environment but are lacking details of the causal mechanisms and factors. This is echoed in the impact category framework presented by Tzilivakis et al. (2024), which identified key metrics for activities, effects and outcomes for a range of impacts. For example, for climate regulation, the metrics include measuring activities that emit GHGs; modeling the effects of emission, such as global warming potential; and measuring outcomes such as changes in climate and consequent impacts on the health of humans, habitats, and species populations. Similarly, for pollination regulation, metrics include measuring land management practices and activities that maintain or increase habitat suitability for pollinators, measuring the effect of those practices on pollinator species populations, and measuring outcomes such as crop yields (dependent on pollination).

A key phenomenon that has been a causal factor for environmental issues is externalization, that is, where issues are not accounted for in decision‐making processes (e.g., because they are not valued by the market). Agricultural production presents classic examples of externalization where, for example, the costs of air and water pollution (and associated impacts, e.g., eutrophication, drinking water quality, climate change) arising from the application of nutrients and pesticides have not been part of the economics associated with the decision‐making processes for crop nutrition and protection (Clapp, 2015; Folkens et al., 2020). The result is that decisions are made that have unintended consequences such as environmental impacts. For example, the creation of the Common Agricultural Policy (CAP) in Europe was driven by the need to ensure food security with a single focus and objective of maximizing yields from agricultural land. The intensification of production that the CAP encouraged led to numerous environmental impacts (e.g., water and air pollution, declines in wildlife and biodiversity) as these were external to decision‐making processes. The CAP has evolved, and environmental objectives are now central to the policy and its instruments, albeit many environmental issues remain. It is now recognized that land use must be multifunctional in the services it provides, that is, not just food production, but a multitude of other ecosystem services. Sustainable food production requires a holistic perspective that integrates multiple objectives into decision‐making, and data are needed to support this. The absence of data risks externalization, and incorrect or biased data risks incorrect decisions within the context of an evolution to sustainable food systems.

The environmental issues covered, the metrics, and the data used are highly variable between different uses of the data. Some are single‐issue focused (e.g., GHG emissions and climate change, or biodiversity), while others aim to cover a wider range of issues. With respect to the latter, many environmental assessments (including the EU Product Environmental Footprint [PEF] methodology) have adopted the impact categories used in Life Cycle Assessment (LCA) (e.g., Hauschild et al., 2011). These include climate change; ozone depletion; ionizing radiation; photochemical ozone formation; particulate matter; acidification; human toxicity and non‐cancer effects; human toxicity and cancer effects; freshwater eutrophication; marine eutrophication; terrestrial eutrophication; freshwater ecotoxicity; land use; water use; resource use, mineral, and metals; and resource use and fossils. When LCA was first presented as an environmental assessment methodology, its holistic perspective was groundbreaking compared to previous approaches. However, in some respects, it has not kept pace with our expanding perspective of environmental impacts and ecosystem services, to the extent that it does not go far enough, with some significant weaknesses both in terms of issues covered and the metrics used. For example, LCA impact categories tend to be limited to those that are emission‐based and/or regional/global impacts, with biodiversity‐related impacts being a known weakness (and many of the metrics used are midpoint effects/impacts, rather than endpoint impacts and outcomes. This is echoed in the ISO 14040 standards for LCA (ISO, 2006; Winter et al., 2017) (and a key issue for sustainable food systems), where endpoint (damage) characterization is an optional step (not mandatory). This largely stems from LCA being a data‐driven framework, and environmental outcome metrics are more difficult to measure.

There are other environmental issue classification systems that take a different and/or more holistic perspective. For example, various ecosystem services classifications (e.g., Haines‐Young & Potschin, 2018). Ecosystem service frameworks are by design utilitarian and focused on the goods and services the ecosystems provide to humans, and although there are elements which focus on biodiversity within those services, they are anthropocentric and thus do not directly account for the needs of wildlife species, albeit with the acknowledgement that biodiversity generally is important for many goods and services (Macfadyen et al., 2012; Muradian & Gómez‐Baggethun, 2021; Schröter et al., 2014).

Tzilivakis et al. (2024) attempted to provide an overarching environmental impact classification framework that encompasses the LCA impact categories and ecosystem services (for humans and wildlife) to provide a more holistic approach to understanding the environmental impacts of food production systems. This framework identified 19 broad impact categories classified into four groups following the four Earth spheres (atmosphere, biosphere, geosphere, and hydrosphere) and identifying the end receptor (human and/or wildlife). It also identifies the key metrics available with respect to activity, effects, and outcomes. With respect to the latter, this includes identifying where the metric draws upon primary (measured) data and/or where secondary (modeled) data tend to be used.

In an ideal world, we would gather primary data for all environmental outcomes and the associated midpoint effects and causes (activities) of those effects. This would not only provide information on the state of the environment but help diagnose issues and devise interventions to address environmental problems. However, gathering primary data on activities, effects, and outcomes is not always practical and too costly to undertake, especially for environmental effects and outcomes. Where this is the case, secondary or modeled data are often used. Secondary data include data from similar systems and/or scenarios as a proxy or using models to calculate environmental effects and outcomes based on primary data that has been directly measured. The latter involves using a known scientific relationship (model) between land use activities and environmental effects and outcomes. In some instances, the scientific evidence is robust and established; in other instances, there is more uncertainty. Additionally, when primary data on activities are limited, then only broad‐brush estimates can be made, increasing uncertainty and decreasing reliability.

Differing perspectives also influence the choice of data used. For example, taking a product‐oriented perspective for carbon accounting, pre‐farmgate emissions, which are usually the largest for food products, are categorized as part of the background system and/or as Scope 3 emissions; that is, they are outside the direct control of the assessed entity, for which secondary data are often assessed to be adequate. However, from a farm‐oriented perspective, pre‐farmgate emissions and impacts are considered part of the foreground system and/or Scope 1 emissions, for which primary data should be sought and used. Secondary data are often averaged global data that are not always relevant to the scenario they are being used for. This can be concerning for some products, especially when coupled with omitted impacts. For example, grass‐fed beef production systems will have lower GHG emissions than other beef production systems but could be assessed to have equal emissions if a global average value for all beef production is used (Desjardins et al., 2012; Gerber et al., 2015; Lynch, 2019; Ritchie, 2020). Additionally, grass‐fed beef production systems can also have environmental benefits such as soil health, water quality, and biodiversity (Bragaglio et al., 2020; de Vries et al., 2015; Jackson, 2022; Mondière et al., 2024; von Greyerz et al., 2023). Therefore, there is scope for this to be misleading for consumers and potentially business‐damaging for the farms who are working to produce beef with a lower environmental footprint (and not helpful within the context of a transition to sustainable food systems). Moving away from the reliance on secondary data will not be easy, as it will take time to produce the primary data from individual farms and have the data handling procedures in place to move the data up the supply chain to be analyzed, interpreted, and utilized. However, as more primary data do become available (via some of the opportunities outlined in Section 3), it is important that this is utilized (rather than using more generic secondary data) to gain a more reliable picture of the sustainability of food systems and to understand where further action and evolution may be needed.

Within the context of ecolabeling, the review by Tzilivakis et al. (2024) found that food product‐based ecolabels tended to have significant omissions of biosphere‐related impacts, and while there was better coverage by farm assurance‐based schemes, these were largely based on practice‐based metrics and not measured environmental outcomes. Biosphere‐related impacts are difficult to measure. Some farm assurance schemes encourage measurement of habitats and species on the farm with the intention of tracking improvement in this over time; however, the outcomes are not part of the requirements for certification. Some LCA impact categories potentially cover aspects of biodiversity but often in proxy or indirectly, such as eutrophication and ecotoxicity, and are not site‐specific. The PEF is looking at including a 17th indicator to include biodiversity in LCA, but it is unclear yet how this will be done and what the timeline is for its inclusion. Similarly, there have been efforts to develop novel metrics to integrate biodiversity into LCA, but none have become established and tend to involve proxies rather than employing outcome‐based metrics (BES, 2025; Lindner et al., 2019). Given the global biodiversity crisis, the lack of biosphere‐related metrics is a significant issue for the transition to sustainable food systems.

#### 2.1.3 Interpretation

Making data meaningful is also a key challenge. This involves interpreting, transforming, and/or communicating the data to support each end purpose (and ensuring data are not misinterpreted). On a basic level, interpretation simply involves categorizing the data and/or presenting them on a relative scale to differentiate a range of options or scenarios. For example, within an ecolabeling context, some schemes score the performance of a product (for single or multiple impacts) relative to other products within a population, thus identifying those which perform better than others (e.g., products may be categorized A to G, high to low). However, this is a relative scale and does not interpret the impact values within the context of the sustainability significance of the impact. Additionally, in some systems, perverse effects can occur; for example, if the boundaries for the A to G categories are set based on the best and worst performing products, flooding the population with higher or lower performing products can change the categorization of an existing product (up or down) with no change in the environmental impact of the product.

To overcome the limitations of relative scales, normalization can be employed to express the impact values relative to a benchmark. For example, GHG emissions can be expressed as a percentage of the average annual per capita emissions in Europe (Sala et al., 2016, 2017). This places the value in the context of other societal activities, but it does not interpret the impact values with respect to their significance. For example, within the context of a transition to sustainable food systems, a key question is what do the data mean in relation to sustainability, and are the values high, moderate, or low; good, bad, or indifferent; sustainable or unsustainable? Such interpretation is strongly linked to (and limited by) our level of scientific understanding of different issues, and as such is an inherently complex issue to address. It is also linked to societal values and priorities, as sustainability encompasses socio‐economic objectives alongside environmental ones. When data are used for different purposes, there is scope for different interpretations, which only serves to blur the picture and increase the scope for contradictions in the data ecosystem.

A common issue with all environmental assessments is whether data can be meaningfully aggregated (e.g., to reduce the amount of data and/or simplify data to aid communication). The inherent risk is that important details are lost during the aggregation process and the picture presented is consequently incorrect and/or missing important information. For example, if a product scores +10 for climate change and −10 for eutrophication, aggregation could result in an overall score of zero, implying the impact of the product is environmentally benign, which is not correct. This is a particular challenge for ecolabeling, where there is a need to convert all the environmental impact data associated with a product into a format that can be simply communicated within the confines of a small label, and for policymaking, where there is often a demand for simplified data or interpretations.

### 2.2 Technological, commercial, and social challenges

The second set of challenges within the data ecosystem relates to what is technologically possible, commercially realistic, and the willingness of socio‐ecological stakeholders, especially farmers. Farm margins are being continually squeezed, and with the changes in farm policy, fluctuations in fertilizer and fuel prices, and challenges with weather and climate change, this situation is not improving (Donnellan et al., 2026; Kuhmonen & Siltaoja, 2022; Putri, 2025). Therefore, any farm‐level intervention needs to be considered carefully, particularly where the cost burden falls, how much farmer time would be involved, and if the benefits are enough. These are some of the barriers encountered when introducing new technologies to farms. Others include the maturity/verification of the proposed technology and ease of use.

The collection, storage, movement, governance, and interoperability of data have been significant issues for many years, generally for agricultural data and more so for environmental data. It needs to be as easy and seamless as possible, but we are still faced with a fragmented framework (due to different demands from different end users and systems operating in the marketplace). Connected to this is the willingness of socio‐ecological stakeholders to engage with both data collection and data sharing, often due to the cost (time and money) and a distrust in how the data will be used.

Data collection for environmental impacts is a challenge for farmers (Antonioli et al., 2024). These activities are often time‐demanding, require expertise they might not have, and use equipment, which is costly to buy and/or requires training to be able to use. The benefit to the farmers for doing the data collection might also be difficult to see, particularly when several sources of data are needed together to build a picture of what is happening on the farm to enable decision‐making in response to it. These issues are exacerbated when considering small‐scale farmers and those operating in developing countries.

Data collection for activities tends to be easier than effects or outcomes because the data are more readily available within a business (Robling et al., 2023). For example, although it can take time, farmers can easily collate data from their records (e.g., fuel use, tractor hours) to put into a carbon calculator. However, measuring gaseous emissions directly is more challenging (e.g., measuring methane emissions from cows, or post‐tillage emissions from soil). Soil sampling and analysis can be done with low level technology, but it provides a snapshot in time and only of the immediate area where the sample is taken, as soil characteristics can vary across a field, so it would be labor intensive to do a thorough screen, with the results not necessarily compensating for the time cost (albeit the technologies for collecting accurate data soil are rapidly evolving—see opportunities below). With respect to biosphere‐related impacts, measuring impacts and outcomes is scientifically challenging. Making observations of species present can be time‐consuming, and for the data to be meaningful, it needs to be collected systematically, spatially, and temporally; species identification often requires expert knowledge; and deriving a meaningful interpretation of the data within the context of wildlife management and biodiversity goals can be problematic.

A long‐term common problem with data handling is interoperability between different software applications (Basir et al., 2024). Many devices, management tools, and databases contain useful farm data but are unable to communicate the data in the same format between them, which can increase the difficulty in data handling and often burden the farmer with repeat input of the same data to different tools, and confusion through multiple tools doing the same task coming out with different results from the same data. For example, a review by ADAS (2023) of six carbon calculators used by farmers highlights the issue of averaging data for livestock over a year to cover the animals that come on and off farm to get emissions ratings. This led to discrepancies in calculator outputs, which would cause problems if the farm used a different calculator at a later point, or when comparing farms that have used different calculators. Farmers are therefore left with not knowing which to trust. It is proposed that this could be remedied through more granular data input. Due to legal requirements for traceability, particularly in cattle systems, the data needed are already collected and stored by the farmer and would produce much more accurate calculator outputs if it could be used as the data input source. However, largely the data are collected in devices which are incompatible and would require separate input into the calculator. This gets more complex for other livestock systems where individual animal data are not captured.

This highlights another key challenge of duplication of effort to collect and enter the same data for different purposes. This is a well‐known issue that has existed for decades (including beyond the digital realm with duplicated paperwork). Doing anything more than once presents a significant barrier to undertaking a task and/or the adoption of new systems and technologies. That said, it is also important to identify where data generation and collection activities are genuine duplication and where they are complementary. Sometimes different data needs to be collected on the same issue but for different purposes, as highlighted above with the example of livestock numbers and types which are required for livestock movement reporting, carbon calculators, or ecolabeling. Thus, it is important that this is understood where this is the case to avoid the assumption that it is duplicated effort.

A further challenge is that of data sharing, particularly concerning data permissions, protection, and control. This is where data are only utilized and passed on to those parties for whom permission has been granted. The movement of data along the supply chain can be contentious due to the uncertainty or unregulated nature of data ownership. Farmers are not always asked permission to share their data, or are not willing to provide their permission, especially if the benefits to them doing so are not a factor in the transfer (Zhang et al., 2021). Consequently, farmers are often concerned about sharing data (Kaur & Dara, 2023). That said, it is often assumed that farmers are unwilling to share data or only willing if it is made mandatory, but the opposite is often the case, especially when the potential benefits and risks are made clear (Van der Burg et al., 2021).

## 3 OPPORTUNITIES

There is little doubt that there are many challenges, but there are also many opportunities within data ecosystems. These include advances in science, technology, and processes that can enhance and advance the data ecosystem to provide a more detailed and reliable picture of agroecosystems. The opportunities broadly fall into those tackling (i) data collection; (ii) data storage, movement, and sharing; and (iii) interpretation.

### 3.1 Data collection

In recent years, there have been numerous advances in science and technology that have the potential to increase and enhance the collection of environmental data (including activities, effects, and outcomes); some of these are operational while others are on the horizon.

#### 3.1.1 Sensors on the ground

First, there are sensors to measure emissions of pollutants (Seesaard et al., 2024). For example, different types of GHG flux meters. Most of these are site‐specific, as they can only be in one place at a time and measure the local environment there. Flux towers (e.g., CEH, 2025) can measure the continual gas exchange between the ground and the atmosphere for large areas but may not supply the level of detail required for some applications. There are also soil sensors that can monitor a range of soil properties (moisture, temperature, pH, nutrients, conductivity), giving an indication of the soil health status. Coupled with other soil tests such as organic matter content, density, and fauna presence including earthworm counts, these can advance understanding. Actionable activities can then be carried out in response, for example, addition of fertilizer or adjusting irrigation rates. If measurements are taken, they can be used to baseline conditions on the farm at a particular time, and monitored subsequently to provide an opportunity to observe change over time, which is particularly useful when farming methods are changed, and progress is wanted to be understood. There is also the Internet of Things, which provides a network of different devices on the farm such as sensors, through the internet so that they are connected to each other and can be accessed by the farmer. If these devices and the data they are creating are fed through artificial intelligence (AI) programs, then the outputs could be even more valuable (provided they are reliable). All these options are providing opportunities to better measure emissions, and potentially environmental outcomes, and thus improve the picture needed to aid the transition to sustainable food systems

Technologies are also being developed to facilitate measuring biodiversity, which as outlined above is a key weakness in the environmental data system for food production. Examples of these are bioacoustics (particularly for insect pollinators), cameras with AI algorithms enabling them to capture insect or animal images and identify them (Chiavassa et al., in press; Kissling et al., 2024), and other technologies as overviewed in MacWilliams et al. (2024). However, they are not yet in common use, and as with many other technologies for measuring impacts on farm, they produce large amounts of data which need interpreting effectively to be useful, although developments in AI can support this. There is also environmental DNA (eDNA) sampling and detection, which has improved in accuracy and usefulness over the last 20 years. It can now be used effectively to detect the presence of organisms in the environment, particularly informing biodiversity studies. Different sample types can be used such as soil and water. The sequencing methods employed can allow for detection of multiple species at the same time but are limited, as they cannot tell which organisms are dead or alive or still present in the sampling area. eDNA studies can now be carried out by trained citizen scientists, and there are several companies operating services for eDNA, so it is much more accessible than previously and can be another tool for farmers to use to understand the environmental impacts on farm, especially biodiversity monitoring. In the United Kingdom, Tesco (2025) has been exploring the use of eDNA, ecology surveys, and bioacoustics data using the latest AI listening technology to find ways to baseline and monitor biodiversity in Tesco's supply chain.

#### 3.1.2 Citizen science and apps

On a simpler level, there are also many advances within the realm of citizen science, especially with respect to monitoring biodiversity with apps like iNaturalist (2025) to aid identification of species using photos and sounds captured on mobile phones. The EU H2020 Framework Project (2025) also encouraged the use of citizen science and associated tools to monitor biodiversity on farms and/or provide guidance of sampling protocols to gather data in a more systematic way to enable trends to be observed reliably over time. Additionally, in the United Kingdom, work commissioned by Natural England (Williamson et al., 2024) has explored farmer participation and engagement in citizen science initiatives and environmental data collecting and monitoring technologies. The Framework Project has also developed a new decision support tool, the Farmland Ecosystem Assessment Support Tool (FEAST) (AERU, 2025), that provides tools to log wildlife species observations, import observations from the Global Biodiversity Information Facility (GBIF, 2025), and visualize these on a map. These can then be combined with assessments of habitat suitability for a range of species or species groups that accounts for landscape connectivity and habitat complementation to provide a thorough assessment of relative strengths and weaknesses in the landscape and thus help identify options to enhance the landscape to meet biodiversity objectives.

#### 3.1.3 Remote sensing

Taking a higher perspective, there are also many advances in remote sensing technologies that have the potential to generate environmental data (Wang et al., 2025). Remote sensing can provide a picture at a greater scale for several impacts and metrics, including soils, through satellite imaging (e.g., Sentinel Earth observation) and drone imaging. This gives a large‐scale overview of a farm area and the wider landscape, as well as more detailed field‐level scans. These methods can generate large amounts of data that need handling and interpreting. If paired with ground‐truthing, the picture can be further verified, albeit adding time, cost, and interoperability challenges. The learnings and insights that can be gained from using remote sensing are valuable to boost farm productivity as well as providing information which can be used for environmental impact monitoring and outcomes assessment.

#### 3.1.4 Business opportunities

Finally, there are business opportunities if farmers take up more data collection technologies, across a wider range of environmental impacts. For example, it could provide the data needed to satisfy Scope 3 emissions reporting demands of their customers or allow entry into nature credit markets. Additionally, and perhaps more fundamentally, if a farming systems change is what is needed to repair our food system, integrating these technologies in a commercially viable way will be crucial to understanding the best and most environmentally beneficial methods of farming, and encourage widespread adoption.

### 3.2 Data storage, movement, and sharing

Once data have been generated, it needs to be stored, moved, and shared, which presents challenges in terms of interoperability, data protection, and governance. The seamless movement from data sources to different applications and end users has been a goal for many decades (in many sectors). In recent years, this is reflected with the emergence of the Findable, Accessible, Interoperable, and Reusable (FAIR) data principles for scientific data (Wilkinson et al., 2016), which are increasingly applied to management and supply chain systems including the agricultural sector (Top et al., 2022). In the food supply chain, in the past, it has been driven by specific issues, especially those associated with food safety and traceability (e.g., FIIN, 2025), and similar challenges exist for environmental data. Interoperability will be key such that the data can be collected, stored, processed, and passed on through different software and devices but still have the same meaning and end up in the same format such that when data users receive them, the data can be utilized in the onward supply chain. Progress in this space is rapidly evolving. Urdu et al. (2024) outline several examples across the food supply chain where advances are being made in interoperability using a range of technologies, and some are focused on environmental data (e.g., OpenSC, 2025; Regrow, 2025), albeit often only tackling single issues (carbon footprints). Software to achieve full interoperability, especially for a broad range of environmental issues, is still lacking, so further innovation is needed in this space. Interoperability is also strongly connected to data governance structures and protocols; thus, advances may go hand in hand with frameworks to manage and govern data.

With respect to data protection and permissions, several countries have drawn up Codes of Practice, including the EU (COPA & COGECA, 2020), and there are initiatives such as the Open Ag Data Alliance (OADA, 2025). These can be utilized when creating contracts with farmers where data will be shared, to specify who can receive it and how widely it can be shared, so the farmer does not lose out on any benefits brought about by the data flow. There are exemplar systems that are doing this but are not yet widely used or suitable to every purpose. In the United Kingdom, the Agriculture and Horticulture Development Board (AHDB) has also seen the need for an improved data ecosystem, which puts more of the data ownership control to farmers who are generating the data and is easy for them to use (AHDB, 2024). They plan to start with the carbon calculators before tackling more of the environmental impacts. Currently, farmers have the choice of whether to account for their carbon emissions and will do so using one of a range of carbon calculators, which is causing inconsistencies in data output. Recently, three of the major carbon calculators used in the United Kingdom have formed a coalition, to work together to harmonize their approach so that farmers still have a choice for which system to use, while ensuring that the outputs are more comparable. This is a good step forward and could provide a model for other environmental impact calculators. In a similar vein, there are efforts to harmonize methodologies for product‐based LCA (Cicek et al., 2024; FDTP, 2024; OECD, 2025), and tools to facilitate the exchange of farm data are under development (e.g., AHDB, 2026).

### 3.3 Interpretation

The final set of opportunities is with respect to interpretation of data for different purposes within the context of sustainability (tackling the interlinked challenges of normalization and aggregation).

#### 3.3.1 Normalization

Normalization remains a key challenge. Scientific understanding is growing, and the data ecosystem needs to reflect that understanding when data are interpreted. Progress is being made, but there is little doubt that this will continue to be a challenging issue to address. For example, the planetary boundaries approach (Rockstrom et al., 2009) attempts to interpret whether an activity has an impact that lies within the capacity of the planet to handle it and still be healthy, or whether it exceeds the limit and needs remediation. For example, Sala et al. (2017) present GHG emissions per capita as a means of normalization and cite a value global normalization factor 8400 kg carbon dioxide equivalent (CO_2_e) per capita (23 kg CO_2_e per capita per day), which provides a useful benchmark to compare emissions. The planetary boundaries value is 2.7 kg CO_2_e per capita per day (Sala et al., 2016), which clearly shows we are living beyond capacity of the planet, illustrating how normalization can greatly enhance interpretation. However, the planetary boundaries approach has only been established for nine impact categories; thus, it does not yet cover all impacts. Even with the recent updates (Richardson et al., 2023), it only provides a narrow perspective and is not holistic enough to determine sustainability (e.g., Mace et al. [2014] highlight that adequate planetary boundaries do not exist for biodiversity, which is a key issue for sustainable food systems). It does however recognize the need for this type of interpretation, that is, placing environmental data within the context of targets that society needs to achieve for sustainability.

#### 3.3.2 Aggregation

In many instances, to aid decision‐making, there is a demand to simplify data, often by aggregation. The key to finding solutions that work is understanding the level of aggregation that aids decision‐making while providing enough detail for a robust and reliable picture. Boiling down the environmental impacts of food production to a single number does not provide a meaningful value to support decisions for sustainability. An environmental profile can be more valuable and provides a richer picture. For example, in the United Kingdom, work has been undertaken in recent years to develop a national pesticide load indicator (PLI) (Rainford et al., 2023; Tzilivakis et al., 2026) to support the development of policies for the sustainable use of pesticides. In the past, the amount of pesticide was used as an indicator of the environmental pressure posed by pesticides. However, this fails to account for the environmental properties of pesticides and their likely impact. The United Kingdom has adapted a Danish approach (Kudsk et al., 2018) but decided that an aggregated metric (as used in Denmark) was not useful for policy development and a suite of fate and ecotoxicity metrics is preferable. For example, this allows the identification and avoidance of burden shift, where decreasing the load on one species is made at the expense of increasing it on another. Tzilivakis et al. (2026) show how this works in practice with the example of pesticide use on sugar beer where it can be seen that there was a significant increase in the use of the insecticide tefluthrin as a seed treatment since 2016 (because of the withdrawal of imidacloprid and clothianidin, which are toxic to pollinators). Tefluthrin is highly toxic to aquatic fauna and flora species, so there has been a burden shift from pollinators to aquatic flora and fauna. The UK government has now adopted the PLI as the basis for a new national target to reduce load across all 20 metrics by 10% between 2018 and 2030 (Defra, 2025). There is, however, no normalization, so it is difficult to contextualize what a 10% reduction in load means with respect to sustainability. It is nevertheless a step in the right direction for providing a more holistic picture to aid the evolution of sustainable food systems.

Striking the right balance for aggregation to aid decision‐making and achieving consensus on interpreting the data are key aspects for generating a clear and consistent picture that will help facilitate the evolution of sustainable food systems. Aggregation and normalization to aid interpretation are not a new phenomenon, but the opportunities and our capacity to address these issues are growing as our science and technology evolves.

## 4 CONCLUSIONS

Data on the environmental impacts and performance of food production systems have undoubtedly grown in recent years; we have a better understanding and more data on causal activities, environmental effects, and outcomes. The advent of smart farming, the Internet of Things, and utilization of big data are just a few examples that are driving a data revolution and evolution in the industry (De Alwis et al., 2022; Wolfert et al., 2017). However, despite this growth in data, we still often lack what is needed to provide a reliable, clear, accurate, and holistic picture of the environmental impacts of food production from farm to fork. The reasons for this lack of data are multiple and complex. In summary, they include methods of data collection that do not exist or are not established; methods of data collection that exist but are impractical within a commercial setting; and data that are collected at the farm level do not meet the needs of end users (further up the supply chain), are in variable formats, and/or are of variable quality. In the absence of data, some end users rely on secondary sources and/or models to derive a picture of environmental impacts and performance. However, these can also vary in reliability, and models do not exist for all parts of the picture (Deconinck et al., 2023). Thus, questions arise over their adequacy as a substitute for primary data. Data collection and handling technologies are growing at a rapid pace; however, they are unlikely to solve all the challenges overnight, especially within a commercial setting.

The ecosystem of environmental data is complex, reflecting both the complexity of the topic and the various socio‐ecological stakeholders supplying, transporting, transforming, interpreting, and consuming data. Stakeholders are seeking greater agri data collaboration (Birch, 2024), and there are initiatives (such as the LED 4 Food project, WRAP, 2025) that are attempting to standardize and harmonize, but for only one part of the ecosystem and largely for one particular purpose (i.e., food product LCAs). Similarly, there are standardized metrics, such as the statutory biodiversity metric tools (Defra, 2024b), that are providing a common format for a specific purpose. However, there is no overarching framework that covers all environmental data for all purposes. This lack of a holistic perspective presents an inherent risk with respect to developing sustainable food systems. This does not mean that the same metrics must be used for all purposes, as they exist for specific and valid reasons. However, it is important to ensure that they are at the very least drawing upon the same primary data and/or presenting the same picture, even if, for example, that picture is narrowed to just one environmental issue. In many instances, it is about how data are transformed and interpreted for specific purposes. Thus, the challenge is to develop the interoperability tools and technologies that meet the needs of each end user while drawing upon common primary data.

A fundamental question is whether the environmental data ecosystem we have is fit for purpose within the context of facilitating a transition to sustainable food systems. The simple answer is no. If we were to build a data ecosystem from scratch, we would not build what we have today. We are in a position of trying to retrofit an existing system; a fragmented system that has evolved in response to different demands from different users and systems. That said, data systems are not set in stone or predetermined, and there is scope for both data suppliers and data consumers to shape them to meet their requirements (Carletto, 2021). Tzilivakis et al. (2024) also suggest this is possible, for example, by prioritizing primary data from farms (utilizing the good work done by farm assurance schemes), expanding metrics to cover more environmental outcomes, and utilizing effective metrics for data collection. This primary data can then feed into LCA methodologies (which as outlined above often draw upon secondary data), which are broadened to include a wider range of indicators that can be understood by consumers, thereby allowing them to make better purchasing decisions.

Taking a wider perspective, the concept of environmental data supply and demand presented in Figure 1 could be interpreted as being linear and transactional (with supply and demand flows from farmers to various end users). However, farmers are both suppliers and consumers of data, and many data users are also potential suppliers. In combination, there is potential to provide a much richer picture of the food system and its environmental impacts. For example, farmers will have high‐quality primary data on their farming practices and, in some instances, environmental effects and outcomes (e.g., soil health and biodiversity). Government regulators and water companies will have primary data on water and air quality where farms are located. NGOs and the public (via citizen science) will have primary data on wildlife populations observed in various locations. Thus, we have a range of data and metrics covering the atmosphere, biosphere, lithosphere, and hydrosphere (some systematically collected, others more anecdotal), and much of this data is in a digital format. However, it is being collected, stored, processed, and interpreted for many different purposes, using different systems in different formats. This perspective is conceptually illustrated in Figure 2, where the linear supply and demand model presented in Figure 1 has been transformed into a spherical model—a “digital donut.” There is a central supply of data, which everyone contributes to, and these data are then processed and interpreted to meet the needs of different end users. We all want the “jam” in the center, the data that give us the best picture. Thus, we need the digital infrastructure to supply that data to those who need it and, importantly, we are drawing up the same primary data to give us a consistent and reliable picture for all end users.

**FIGURE 2:**
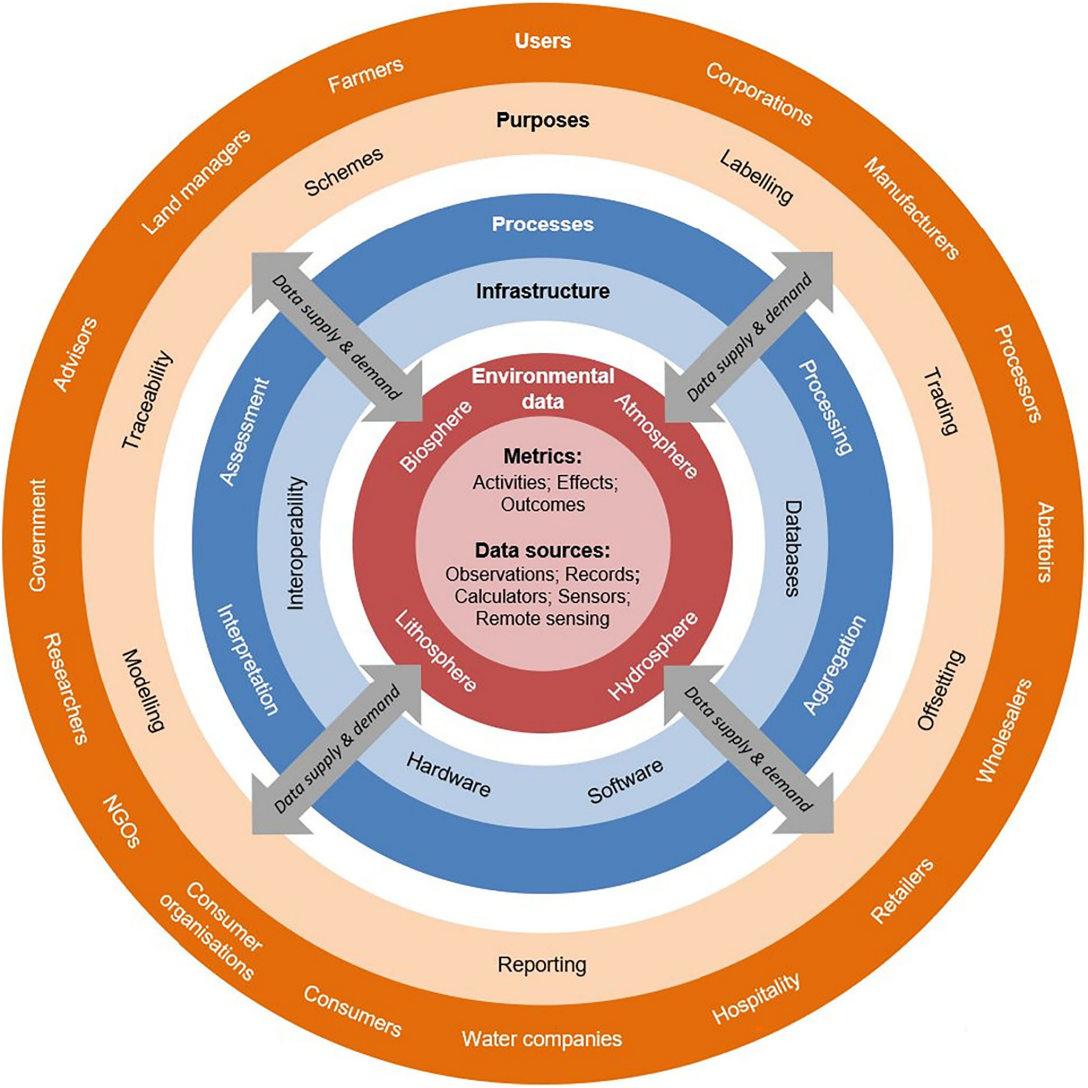
Supply and demand in data ecosystem: A digital donut.

There are still many challenges to overcome for both the collection of environmental data and using it effectively to aid decision‐making and drive positive change. However, these also present opportunities where real change could be encouraged such that national and international food security and sustainability issues are addressed. With the push for more joined‐up thinking, cooperation, and harmonization between the different stakeholders in the food production system, there is progress to come and solutions to be developed to ensure that the important work farmers do to produce food and manage their land to benefit the environment is reliably reflected in the data ecosystem. The improvements made to the data ecosystem will provide a better and more holistic understanding of food production systems, which aids the diagnosis of issues and the identification of solutions that will deliver sustainable food systems.

## AUTHOR CONTRIBUTIONS

**J. L. Taylor**: Conceptualization; formal analysis; investigation; writing—original draft. **J. Tzilivakis**: Conceptualization; formal analysis; investigation; project administration; writing—original draft.

## CONFLICT OF INTEREST STATEMENT

The authors declare no conflicts of interest.
